# Upper Gastrointestinal Tract IrAEs: A Case Report About Sintilimab-Induced Acute Erosive Hemorrhagic Gastritis

**DOI:** 10.3389/fimmu.2022.840916

**Published:** 2022-06-03

**Authors:** Qi Ai, Wen Chen, Yonggui Li, Guoqing Li

**Affiliations:** The Second Affiliated Hospital, Department of Digestive Internal Medicine, Hengyang Medical School, University of South China, Hengyang, China

**Keywords:** sintilimab, immune checkpoint inhibitor, acute erosive hemorrhagic gastritis, immune-related adverse event, upper gastrointestinal tract irAE

## Abstract

**Introduction:**

Immune checkpoint inhibitors (ICIs) have now become the standard therapy for malignancies like non-small cell lung cancer and classical Hodgkin’s lymphoma. ICIs are associated with unique immune-related adverse events (irAEs) caused by dysregulated immune activation. Treatment of lower gastrointestinal (GI) tract irAEs, such as colitis, is more common. However, for upper gastrointestinal tract irAEs, there is a lack of consensus in terms of globally standardized disease classification and treatment guidelines. Here, we report a case of sintilimab-induced acute erosive hemorrhagic gastritis.

**Case Presentation:**

A 54-year-old man with metastatic NSCLC (PT2N2M1 stage IV) underwent treatment with eight courses of sintilimab + bevacizumab, followed by maintenance therapy with sintilimab alone. However, he presented with epigastric pain and melena at the end of the first sintilimab treatment, and the symptoms occurred repeatedly after regular treatment with acute erosive hemorrhagic gastritis. Repeat esophagogastroduodenoscopy (EGD) showed severe hemorrhagic gastritis; symptomatic relief and improvement in EGD images were noted for as long as he was being treated with steroids, methylprednisolone sodium.

**Conclusion:**

As far as we are aware, we here describe the first case of sintilimab-associated acute erosive hemorrhagic gastritis, an upper gastrointestinal toxicity event. Throughout the treatment progression, differential diagnosis, multidisciplinary discussion, and the use of immunosuppressants were instrumental in clarifying the diagnosis and were crucial to the prognosis of the patient and continued treatment with ICIs.

## Introduction

Currently, immune checkpoint inhibitors (ICIs) are a hot topic in the field of oncology research and the mainstay of metastatic malignancies because immunotherapy of malignancies improves the survival of patients in whom conventional therapy has failed (1, 2). Immune checkpoint blockade enhances antitumor immunity by blocking immune innate downregulatory factors such as cytotoxic T-lymphocyte antigen 4 (CTLA-4) and programmed cell death 1 (PD-1) or its ligand (PD-L1). Sintilimab, a recombinant and fully human immunoglobulin G4 (IgG4)-type anti-programmed cell death receptor-1 (PD-1) monoclonal antibody, activates T-cell function by binding with PD-1 and blocking the binding of PD-1 to PD-L1 and PD-L2, thus relieving the immunosuppressive effect, enhancing the immunosurveillance and killing ability of T cells against tumors, and generating an immune response against the tumor (2, 3). ICIs have revolutionized the treatment of metastatic cancer, and although their unique immune-related adverse events (irAEs) in the lower GI tract, such as colitis, are commonly discussed, irAEs in the upper GI tract are discussed sparingly. Furthermore, the clinical manifestations of upper GI toxicity are less well described in the previous literature, and currently, there is no uniform classification standard for ICI-associated gastritis (2, 4–6).

As treatment with sintilimab is becoming more common in immune therapy, cases of sintilimab-induced **upper gastrointestinal tract** irAE are limited. While sintilimab-associated **upper gastrointestinal toxicity events** are rarely recognized, here we report a case **due to sintilimab**-induced acute erosive hemorrhagic gastritis.

## Case Presentation

A 54-year-old male was diagnosed as having lung cancer. He underwent video-assisted thoracoscopic surgery for left upper lung cancer in the Second Affiliated Hospital of the University of South China, and his postoperative diagnosis was polymorphic adenocarcinoma of the left upper lung (T2aN2M0 stage IIIA). Immunohistochemical staining showed the following: CK (+), CK7 (+), TTF1 (+), Napsin A (part+), Vim (+), Syn (−), CgA (−), CD56 (−), P40 (−), CK5/6 (−), and Ki67 (50%+). After the surgery, he was started on the TP chemotherapy regimen, which included the administration of carboplatin (40 mg, days 1–3) + paclitaxel (400 mg, day 1). Four cycles of this TP chemotherapy regimen were administered, with each cycle lasting about 21 days. However, about 178 days postoperatively, he was diagnosed with brain metastases [pT2N2M1 stage IV, epidermal growth factor receptor/anaplastic large-cell lymphoma kinase/ROS1 negative, Kirsten rat sarcoma virus (KRAS) mutation 71.8%, KRAS amplification 2.4, cyclin D1 gene amplification 2.6, cyclin-dependent kinase inhibitor 2A mutation 9.1%, and PD-L1 tumor proportion score 60%], after which he received eight courses (approximately 21 days per course) of bevacizumab (1,100 mg) + sintilimab (200 mg) from 2020 to 2021. In April 2021, he was started on sintilimab maintenance therapy. Bevacizumab was discontinued. About 10 days later, he soon visited our hospital for epigastric pain. He underwent gastroscopy on day 419, which showed squamocolumnar junction (SCJ) blurred, local mucosal hyperemia edema with erosion and opaque white mucus covering the mucosal surface, and localized hemorrhage. A large amount of opaque white mucus was attached to the mucosal surface of the gastric antrum and body, and the gastric mucosa was diffusely hyperemic with spontaneous hemorrhage (**Supplementary Figure 1**). Colonoscopy showed no abnormalities. The patient was treated with oral ilaprazole (5 mg, once daily) and gastric protection with sucralfate oral suspension (1 packet, twice daily). Repeat esophagogastroduodenoscopy (EGD) performed in May showed improvements, and repeat colonoscopy still showed no abnormalities. Then, he stopped the oral drug treatment without permission from the doctors after the symptoms subsided (**Supplementary Figure 2**).

**Figure 1:**
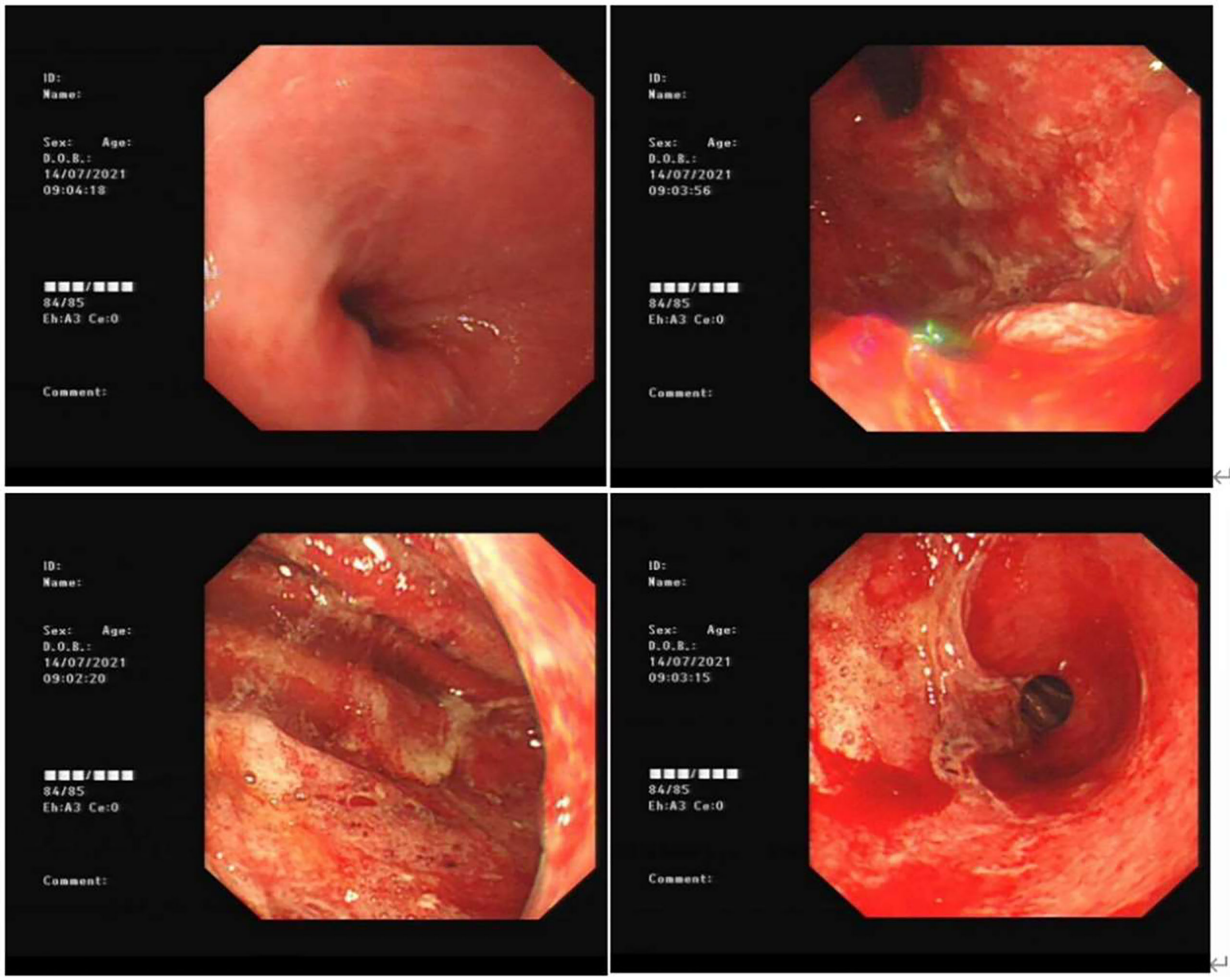
Gastroscopy of acute erosive hemorrhagic gastritis for the first time (day 502). The esophageal mucosa was smooth with good contraction; the whole gastric mucosa was evidently edematous and hyperemic with opaque mucous adhesions on the surface. Diffuse erosion of white moss was seen, and active oozing of blood was extensively observed. The gastric antrum was found to have a necrotic mucosa with exfoliation of large flakes.

After treatment with three courses of sintilimab alone, he had recurrent epigastric pain and black tarry stools, which were worse than before. Repeat EGD showed that the entire gastric mucosa was evidently edematous and hyperemic and showed adhesion of opaque mucus, diffuse erosion of the white moss, and active localized hemorrhaging was extensively observed. The gastric antrum was found to have a necrotic mucosa with exfoliation of large flakes (**Figure 1**). The Carbon 14 breath test was negative, and tests for cytomegalovirus and Epstein–Barr virus revealed a previous infection, but not a current infection. The patient was also negative for tuberculosis immunoglobulin G (IgG) antibodies, HBV, HCV, syphilis antibodies, and HIV (**Supplementary Table 1**). Initially, he was treated with esomeprazole (40 mg every 6 h *via* an intravenous pump), oral rebamipide (100 mg, thrice daily), sucralfate oral suspension (10 ml, twice daily), and rehabilitative new liquid (10 ml, thrice daily, taken orally). However, the symptoms of the patient did not subside and were aggravated even with consuming a small amount of water or other fluids. After regular treatment for acute erosive hemorrhagic gastritis, his fecal occult blood test was still positive, indicating that the upper GI lesion still existed.

**Figure 2:**
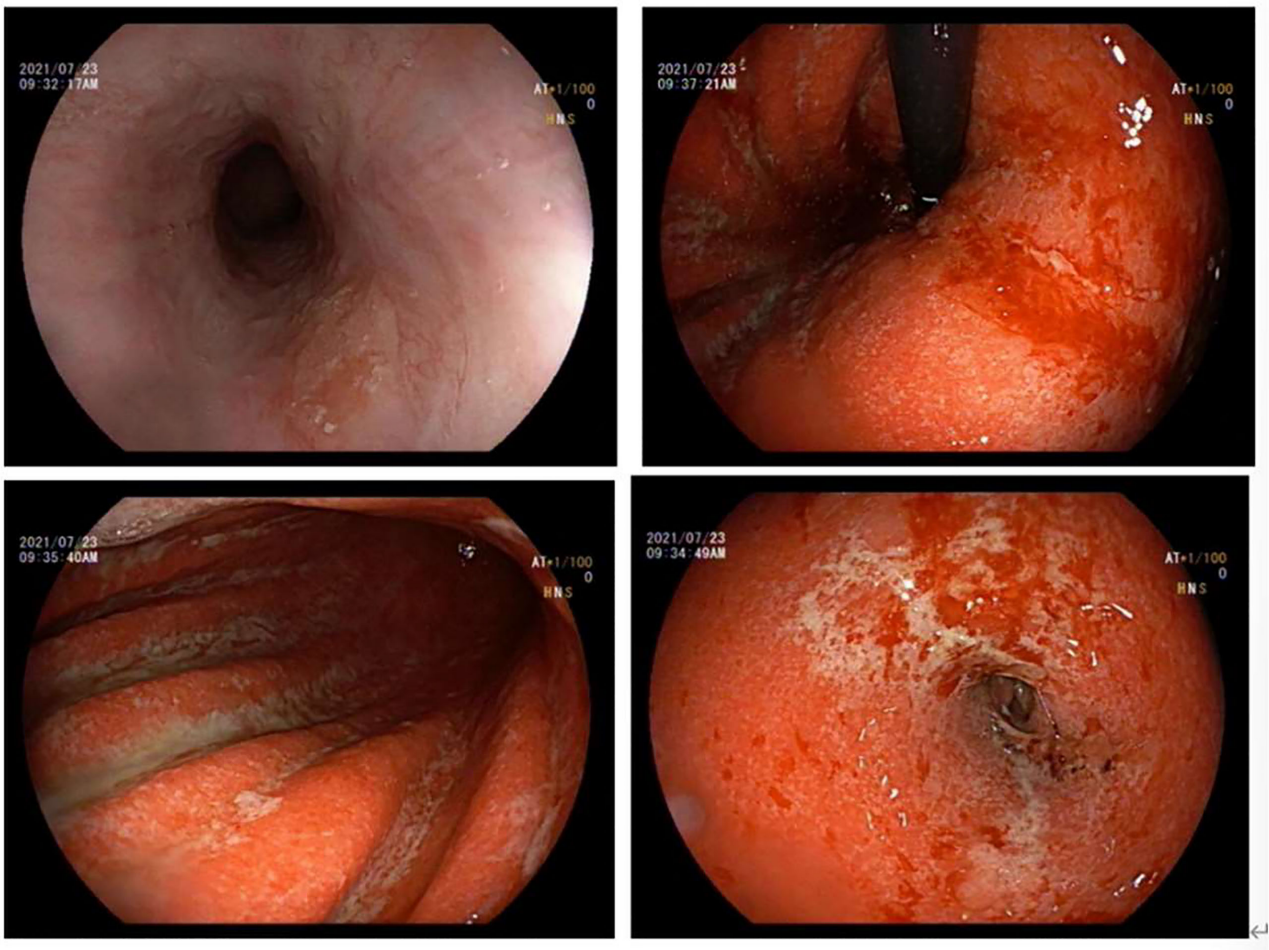
Gastroscopy of acute erosive hemorrhagic gastritis after regular treatment (day 511). The esophageal mucosa was smooth with good contraction, and his EGD showed the whole gastric mucosa was hypermic and edematous, with diffuse erosion of white moss and active oozing of blood. Small flaps of mucosal necrosis and stripping could be seen at the posterior wall of the gastric antrum, which showed a relatively more healed condition than seen in the previous EGD.

**Figure 3:**
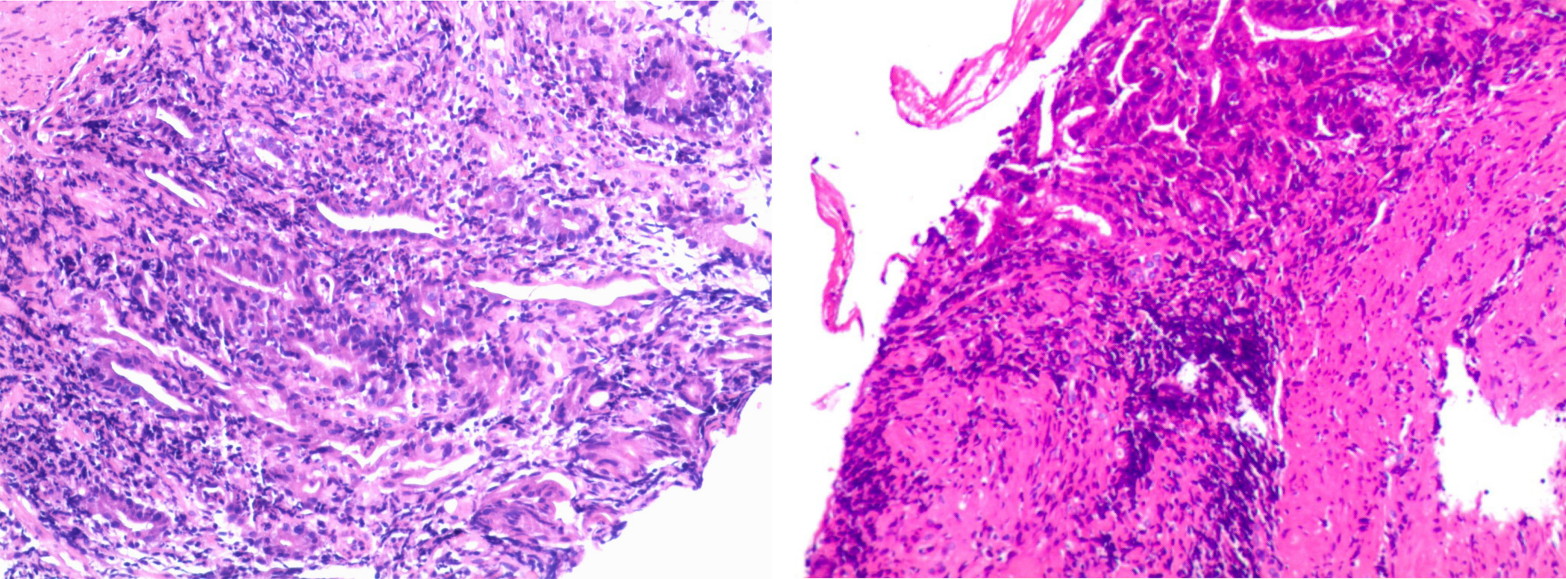
Pathologic findings of the biopsy specimen showed severe atrophic gastritis with erosion, atrophy, and loss of intrinsic glands; focal aggregation of lymphocytes in the lamina propria and mucosal muscular layer; multiple neutrophil infiltration; and the formation of small abscesses.

After three days of the abovementioned regular treatment for acute gastritis, his EGD revealed that the whole gastric mucosa was hyperemic and edematous, with erosion of the attached diffuse white moss and active oozing of blood. Small flaps of mucosal necrosis and stripping could be visualized at the posterior wall of the gastric antrum, indicating a relatively healed mucosa compared with previous EGD findings (**Figure 2**). Pathologic findings of the biopsy specimens from the gastric antrum showed severe atrophic gastritis with erosion, atrophy, and loss of intrinsic glands; focal aggregation of lymphocytes in the lamina propria and mucosal muscular layer; multiple neutrophil infiltration; and the formation of small abscesses (**Figure 3**). Considering that bevacizumab has already been discontinued for almost 2 months, it can be concluded that the current presentation was not an effect of bevacizumab; we confirmed that his gastritis was associated with the immune checkpoint inhibitor sintilimab.

**Figure 4:**
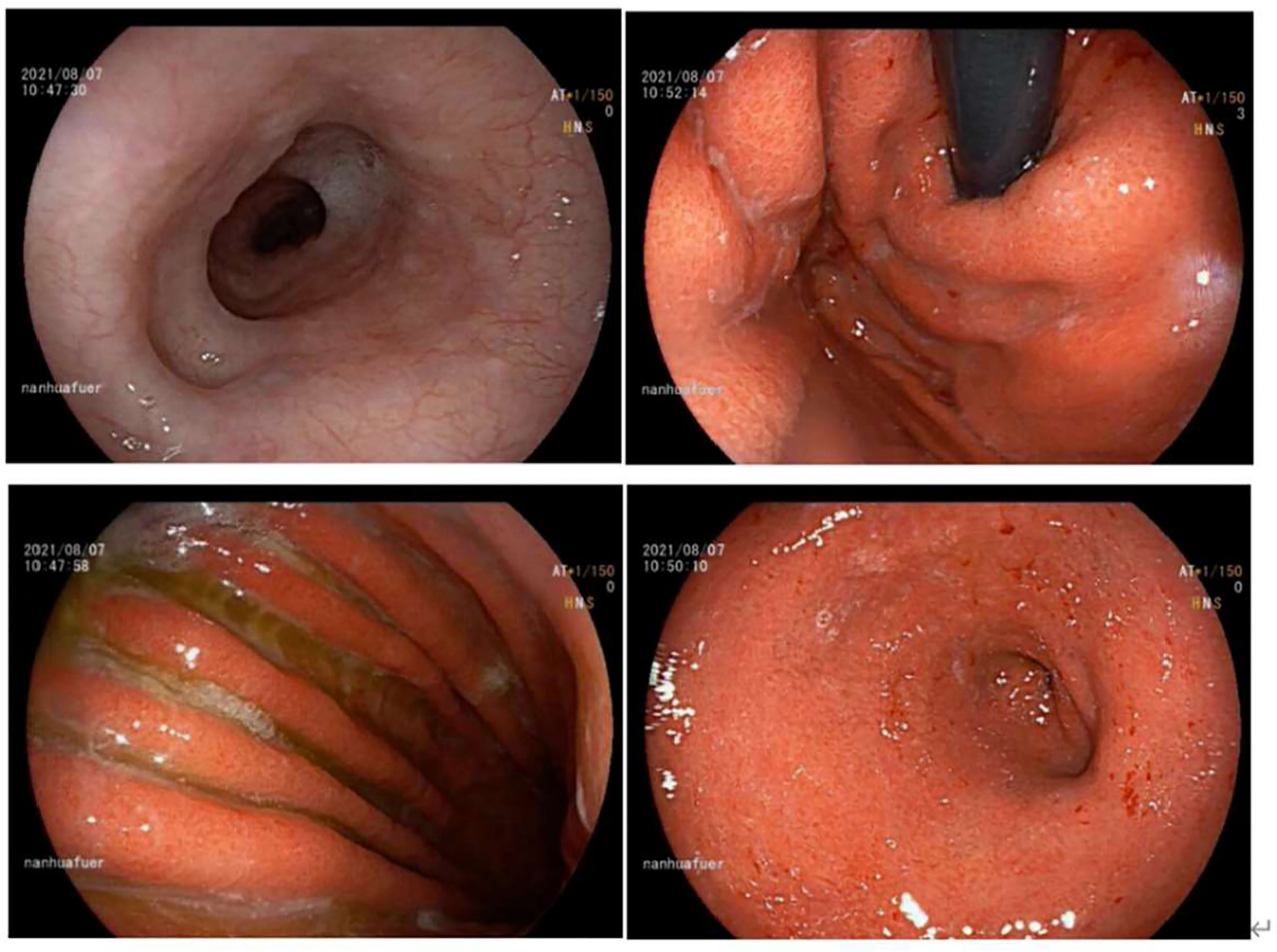
Gastroscopy of acute erosive hemorrhagic gastritis after steriod treatment (day 526) showed marked improvements. The esophageal mucosa was smooth with good contraction. The whole gastric mucosa was hyperemic and edematous, with scattered flaky erosion (particularly in the gastric antrum) and partial bleeding. The gastric mucosa showed yellow–green bile and some white opaque mucus. Compared with the previous EGD findings, the mucosa as seen in this EGD was basically healed.

We suspended sintilimab administration and simultaneously changed the regimen to steroid therapy as follows: intravenous methylprednisolone sodium succinate drip (100 mg, once daily for 4 days; 70 mg, once daily for 3 days; and 40 mg, once daily for 3 days), followed by oral methylprednisolone tablets as maintenance therapy (32 mg once daily for a week; **Supplementary** **Figure 3**). His symptoms gradually subsided after this treatment. Re-examination of EGD on day 526 showed markedly relieved edematous and hyperemic gastric mucosa and no signs of active oozing of blood compared to previous EGD findings (**Figure 4**). The patient began to resume the next course of sintilimab treatment (200 mg, 42 days per cycle) starting in October (day 598). Approximately half a month later (day 613), his EGD revealed that the entire gastric mucosa was hyperemic and edematous with white opaque mucus attachment; however, no erosion, ulcer, or mass was identified (**Supplementary Figure 5**).

## Discussion

Notably, ICIs are becoming an important component in treating metastatic malignancies. However, there are various organ-specific irAEs associated with the administration of ICIs that limit their widespread use, and in patients with severe irAEs, treatment with ICIs needs to be suspended or permanently discontinued; this greatly hampers the continuity of the treatment. Adverse events associated with ICIs are considered to arise after the inhibition of auto-regulatory signals, which leads to autoimmune-like events. There are grading criteria for ICI-associated colitis and diarrhea; although their diagnosis, disease classification, and treatment have already been established, patients with a certain severity may even require ICIs to be permanently discontinued. Thus far, there are no uniform criteria for grading the severity of ICI-associated gastritis, and the reports on ICI-associated gastritis are not only few but also lacking in terms of indications for therapy.

According to the 2021 guidelines of the Chinese Society of Clinical Oncology, the combination of PD-1/PD-L1 inhibitor and platinum-based chemotherapy is the first-line treatment regardless of PD-L1 expression in squamous and non-squamous NSCLC without mutational cancer drive genes. Administering a single PD-1/PD-L1 inhibitor has become the standard second-line option in patients with a PD-L1 tumor proportion score of ≥50% (7). The combination of Sintilimab + pemetrexed or carboplatin has been approved by the Chinese National Medical Products Administration as the first-line treatment of EFGR/ALK-negative, advanced non-squamous NSCLC. The ORIENT-11 study, which was a Chinese phase III study, showed that compared with the chemotherapy group patients, the sintilimab + chemotherapy group patients had significantly longer PFS (median 8.9 months vs. 5.0 months, P <0.00001) and improved ORR (51.9% vs. 29.8%, P = 0.00003). The supplementation of standard chemotherapy (pemetrexed and a platinum-based drug) with sintilimab in Chinese patients having previously untreated locally advanced or metastatic non-squamous NSCLC markedly prolonged their PFS with a manageable safety profile (7, 8). Additionally, the combination of anti-vascular endothelial growth factor (VEGF) agents and immune checkpoint blockade could have synergistic antitumor activity along with favorable tolerance in the tumor microenvironment, and notably, clinical evidence supports that a combination of an antiangiogenic agent and immunotherapy offers increased efficacy (9–11). Herbst et al. evaluated the safety and tolerability of pembrolizumab + ramucirumab in patients with advanced NSCLC and reported that the objective response rate was 30% and the disease control rate was 85% (12). In the IMpower150 clinical trial, the addition of atezolizumab to bevacizumab + chemotherapy significantly improved PFS and OS in patients with metastatic non-squamous NSCLC, and this outcome was unaffected by PD-L1 expression and EGFR or ALK genetic alteration status of the patients (13).

Bevacizumab is a recombinant humanized monoclonal antibody that binds to human VEGF and blocks its biological activity as an inhibitor of tumor angiogenesis. The adverse effects of bevacizumab affecting the digestive system typically manifest as diarrhea, nausea, vomiting, and abdominal pain. When the patient was first diagnosed with acute erosive hemorrhagic gastritis in April, we considered that bevacizumab may have induced the condition. Then, bevacizumab was discontinued, and the patient was treated with **sintilimab** alone as maintenance therapy. However, he was again diagnosed with acute erosive hemorrhagic gastritis two months later, and considering that it had already been two months since bevacizumab was stopped, bevacizumab was not considered to have caused this complication. Notably, the diagnostic process of ICI-related acute erosive hemorrhagic gastritis has exclusiveness; the patient’s colonoscopy showed no abnormalities, which did not support the diagnosis of ICI-related colitis or inflammatory bowel disease (IBD). Additionally, the patient did not have a history of *Helicobacter pylori* infection or non-steroidal anti-inflammatory drug use, and his histopathology findings showed inflammatory cell infiltration, which was identified on the basis of the presence of lymphocytes in the lamina propria. Combined with the strong immunogenicity of PD-1, we hypothesized that his gastritis was triggered by **sintilimab**.

After the diagnosis of irAEs was clarified, the patient was advised to withhold ICI therapy and was started on periodic steroid de-escalation therapy (2, 14, 15). In most cases, acute erosive hemorrhagic gastritis can heal and the bleeding can stop without any intervention. However, in some cases, mucosal erosion may develop into ulcers, which usually respond well to drug therapy. During treatment progression, our patient responded well to steroids instead of regular drug therapy, which can further support **our diagnosis to be correct.**

Previous studies have suggested that the histopathological patterns of ICI-associated gastritis can be divided into three types: chronic active gastritis, lymphocytic gastritis, and focal enhancing gastritis (5, 16, 17). The pathological finding**s**of the gastric antrum in our case show severe atrophy of the gastric sinus mucosa with lymphocytic and neutrophil infiltration, with visibly evident local abscess formation but no signs of apoptotic bodies. Immunostaining identified these lymphocytes as predominantly CD3-positive T cells, CD4-positive helper T cells, and CD8-positive cytotoxic T cells, which is consistent with the pathological findings of ICI-associated gastritis (**Supplementary** **Figure 4**). Given that EGD images have features nonspecific to ICI-associated gastritis, immunostaining may greatly help diagnose upper GI disorders induced by ICIs. Interestingly, the spectrum of clinical presentations of ICI-related gastritis is limited and nonspecific, and endoscopic features range from mild inflammation to severe hemorrhagic gastritis.

Although checkpoint inhibitor-induced gastritis has been reported with PD-1 like nivolumab (18–20) and pembrolizumab (21), to our knowledge, the effects of sintilimab on the upper GI tract have not been described so far. We reported here a rare case of acute erosive hemorrhagic gastritis induced by sintilimab, suggesting a presentation consistent with the potent immunogenic effects shown by other ICIs. A few cases of acute erosive hemorrhagic gastritis caused by ICIs have been reported till date, and our case had more extensive and severe lesions than similar cases reported previously (18, 20–22). Therefore, we believe that this case report not only makes a case for adding a new subtype of ICI-associated gastritis but also provides a reference for future grading and treatment guidelines for ICI-associated gastritis.

Of course, this study has certain limitations: (1) interleukin-17 has been reported to be elevated in the serum of patients with ipilimumab-induced colitis, suggesting that cytokines may be involved in the pathophysiological process of immune-related adverse events; however, we did not perform the interleukin-17 relevant tests for in this case (23) (2). Patients with genetic polymorphisms associated with IBD may be more prone to ICI-associated, gastrointestinal tract-related immune adverse reactions, and it is believed that patients with IBD susceptibility genes should be considered for use in helping diagnosis as early as possible, in which case we did not refine the relevant tests (3). Although early administration of infliximab can improve the prognosi**s**of patients with ICI-related colitis, it has been suggested that the addition of infliximab in patients with severe ICI-related colitis should be considered only when the effect of glucocorticoids is poor at 48 h after administration (15), and furthermore, it has also been suggested that diffuse lesions of ICI-associated colitis should be treated with biologics as early as possible (2, 14). Treatment with infliximab was not considered given the high price of infliximab, the lack of consensus guidelines for ICI-associated gastritis, and the fact that the clinical symptoms gradually resolved with glucocorticoids in this study.

## Concluding Remarks

Taken together, early detection, early diagnosis, and early treatment are critical for irAEs to avoid the associated complications and enable the patient to continue ICIs. Early diagnosis and early use of immunosuppressive agents, such as glucocorticoids and infliximab, in treatment can be beneficial for patients. This case report will help standardize the treatment of ICI-associated gastritis, deepen our understanding of various types of irAEs, and help clinicians make the correct diagnosis and administer treatment promptly.

## Data Availability Statement

The original contributions presented in the study are included in the article/**Supplementary Material**. Further inquiries can be directed to the corresponding author.

## Ethics Statement

Written informed consent was obtained from the individual(s) for the publication of any potentially identifiable images or data included in this article.

## Author Contributions

QA and WC wrote the paper, they have contributed equally to this work. YL provided technical or material support. GL designed the paper. All authors listed have made a substantial, direct, and intellectual contribution to the work and approved it for publication.

## Conflict of Interest

The authors declare that the research was conducted in the absence of any commercial or financial relationships that could be construed as a potential conflict of interest.

## Publisher’s Note

All claims expressed in this article are solely those of the authors and do not necessarily represent those of their affiliated organizations, or those of the publisher, the editors and the reviewers. Any product that may be evaluated in this article, or claim that may be made by its manufacturer, is not guaranteed or endorsed by the publisher.

## Supplementary Material

Supplementary Figure 1 Gastroscopy (day419) showed squamocolumnar junction blurred. Local mucosa was hyperemic and edematous. Flaky erosion was noted, and the surface was covered with white mucus. Oozing of blood was noted. A large amount of white opaque mucus was attached to the mucosal surface of the gastric antrum and body, and the gastric mucosa was diffusely hyperemic with spontaneous hemorrhaging.

Supplementary Figure 2 Gastroscopy (day 449) showed an improved condition than seen on day 419.

Supplementary Figure 3 Timeline of hospitalization and agents used. **(A)** Immunostaining shows CD3 positivity **(B)** CD4+ **(C)** CD8+ **(D)** HE4x10 **(E)** HE10X10 **(F)** HE20X10.

Supplementary Figure 4 IHC **(A–C)** Immunostaining shows mainly CD3-positive **(A)** and CD4-positive **(B)** lymphocytes with CD8-positive cells **(C–F)** Histopathology shows inflammatory cell infiltration based on lymphocytes in the lamina propria. **(D)** Stomach (hematoxylin–eosin, 4x10, bar 100 μm). **(E)** Stomach (haematoxylin–eosin, 10x10, bar 100 μm). **(F)** Stomach (haematoxylin–eosin, 20x10, bar 100 μm).

Supplementary Figure 5 Gastroscopy (day 613): Esophageal mucosa was smooth with good contraction. The whole gastric mucosa was hyperemic and edematous, with white opaque mucous attachments but no erosion, ulcer, or mass.

Supplementary Table 1 Laboratory data Carbon 14 breath test was negative, and cytomegalovirus and Epstein–Barr virus detection showed previous infection, not current infection. Tuberculosis IgG antibody,HBC, HCV, syphilis antibody, and HIV were also negative.
