# Supplementary material for: Upper Gastrointestinal Tract IrAEs: A Case Report About Sintilimab-Induced Acute Erosive Hemorrhagic Gastritis
Source: Front Immunol. 2022 Jun 3;13:840916. doi: 10.3389/fimmu.2022.840916 (PMC9204206; doi:10.3389/fimmu.2022.840916)
Supplement: Supplementary Figure 1 — Gastroscopy (day419) showed squamocolumnar junction blurred. Local mucosa was hyperemic and edematous. Flaky erosion was noted, and the surface was covered with white mucus. Oozing of blood was noted. A large amount of white opaque mucus was attached to the mucosal surface of the gastric antrum and body, and the gastric mucosa was diffusely hyperemic with spontaneous hemorrhaging. [file Presentation_1.pdf]

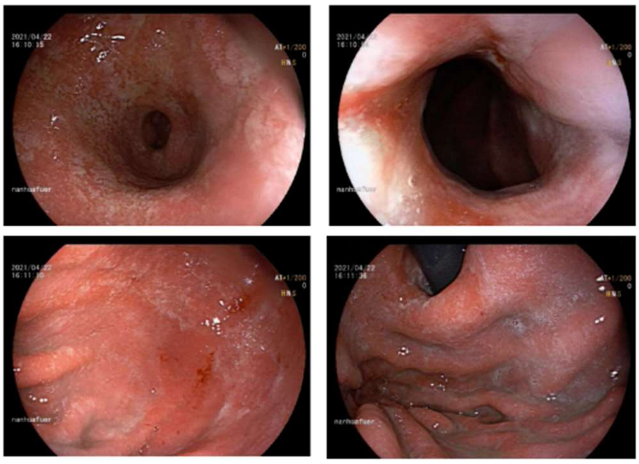

**Supplementary Figure 1.** Gastroscopy (day419) showed squamocolumnar junction blurred. Local mucosa was hyperemic and edematous. Flaky erosion was noted, and the surface was covered with white mucus. Oozing of blood was noted. A large amount of white opaque mucus was attached to the mucosal surface of the gastric antrum and body, and the gastric mucosa was diffusely hyperemic with spontaneous hemorrhaging.

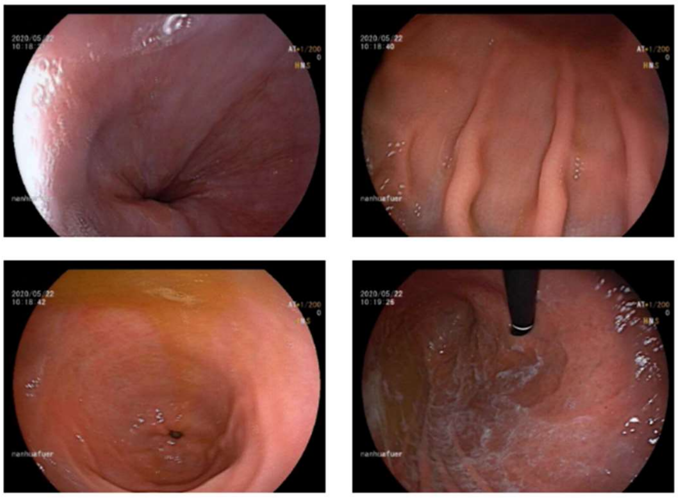

**Supplementary Figure 2.** Gastroscopy (day 449) showed an improved condition than seen on day 419.

| Variable | Reference Range, Adults | Result |
|---|---|---|
| White blood cell count | 4.0~10.0^9/L | 7x10^9/L |
| PCT | 0.0~0.05ng/ml | <0.05ng/ml |
| CRP | 0.0~3.58mg/L | 15.8mg/L |
| Hepatitis B surface antigen | Negative | Negative |
| Hepatitis B surface antibody | Negative | Negative |
| Hepatitis B total core antibody | Negative | Negative |
| Hepatitis B e antigen | Negative | Negative |
| Hepatitis B e antibody | Negative | Negative |
| Hepatitis B DNA (IU/mL) | Not detected | Not detected |
| EBV viral capsid antigen IgM antibody | Negative | Negative |
| EBV viral capsid antigen IgG antibody | Negative | Negative |
| Syphilis antibody（IgM/IgG） | Negative | Negative |
| HIV | Negative | Negative |
| Hepatitis C antigen | Negative | Negative |
| CMV IgM (units) | 0.0~4.2AU/ml | 0.06AU/ml |
| CMV IgG (units) | 0.0~2.0AU/ml | 3.332AU/ml |
| TB-Ab（IgG） | Negative | Negative |
| EBVCA-IgM | Negative | Negative |
| EBVCA-IgG | Negative | Positive |
| EBNA1 IgG | Negative | Positive |
| EB-VCA-IgA | Negative | Positive |
| EA-IgA | Negative | Negative |
| EA-IgG | Negative | Negative |

**Supplementary Table 1.** Laboratory data

Carbon 14 breath test was negative, and cytomegalovirus and Epstein–Barr virus detection showed previous infection, not current infection. Tuberculosis IgG antibody,HBC, HCV, syphilis antibody, and HIV were also negative.

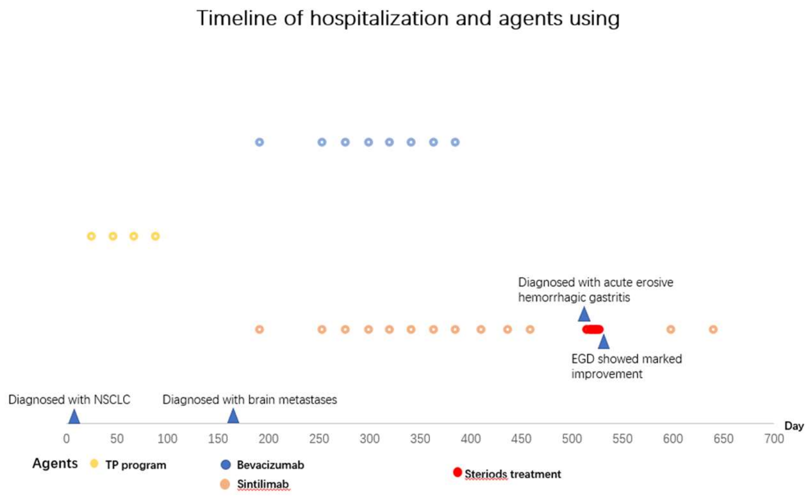


**Supplementary Figure 3.** Timeline of hospitalization and agents used.

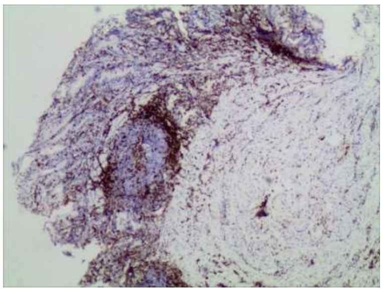

(A) Immunostaining shows CD3 positivity

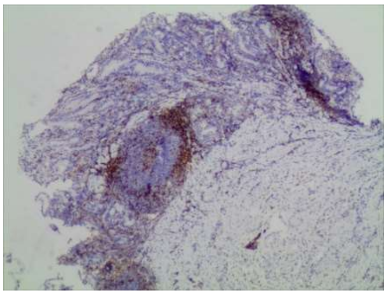

(B) CD4+

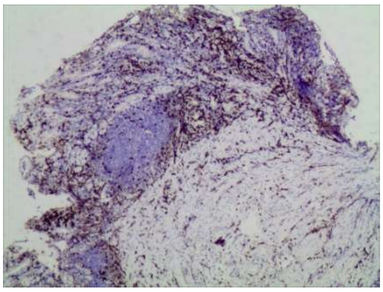

(C) CD8+

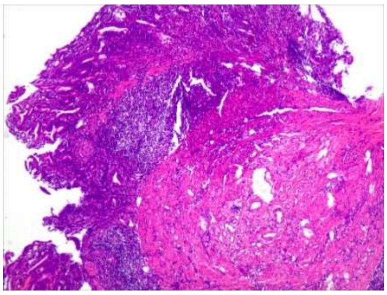

(D) HE4x10

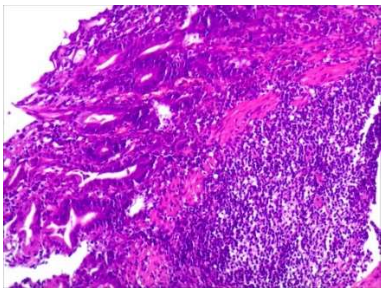

(E) HE10X10

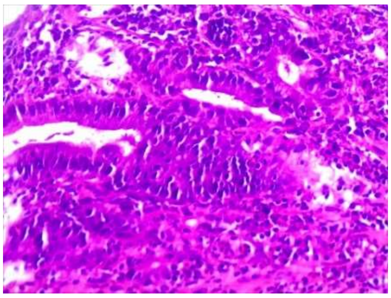

(F) HE20X10

**Supplemental Figure4. IHC**

(A–C) Immunostaining shows mainly CD3-positive (A) and CD4-positive (B) lymphocytes with CD8-positive cells (C)

(D–F) Histopathology shows inflammatory cell infiltration based on lymphocytes in the lamina propria. (D) Stomach (hematoxylin–eosin, 4x10, bar 100 μm). (E) Stomach (hematoxylin–eosin, 10x10, bar 100 μm). (F) Stomach (hematoxylin–eosin, 20x10, bar 100 μm).

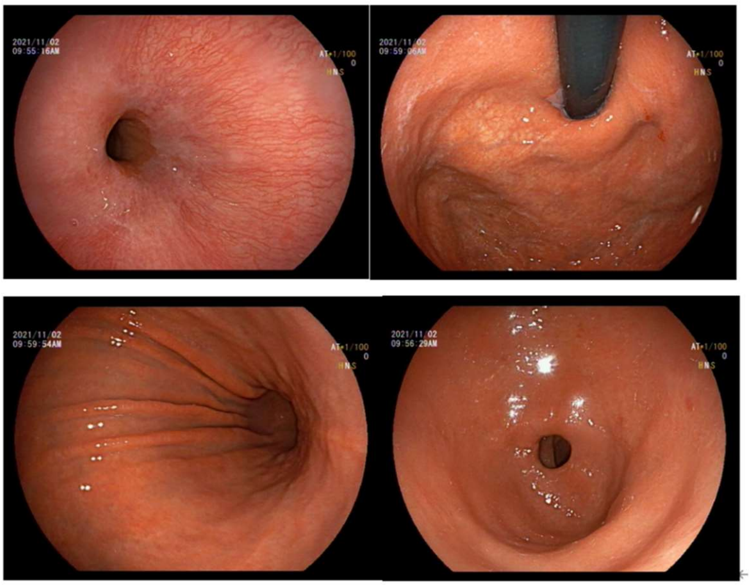

**Supplemental Figure 5.** Gastroscopy (day 613)**:** Esophageal mucosa was smooth with

good contraction. The whole gastric mucosa was hyperemic and edematous, with white opaque mucous attachments but no erosion, ulcer, or mass.
